# Microglial Amyloid-*β*1-40 Phagocytosis Dysfunction Is Caused by High-Mobility Group Box Protein-1: Implications for the Pathological Progression of Alzheimer's Disease

**DOI:** 10.1155/2012/685739

**Published:** 2012-05-08

**Authors:** Kazuyuki Takata, Tetsuya Takada, Aina Ito, Mayo Asai, Manami Tawa, Yuki Saito, Eishi Ashihara, Hidekazu Tomimoto, Yoshihisa Kitamura, Shun Shimohama

**Affiliations:** ^1^Department of Clinical and Translational Physiology, Kyoto Pharmaceutical University, Misasagi, Yamashina-ku, Kyoto 607-8414, Japan; ^2^Department of Molecular Cell Physiology, Kyoto Prefectural University of Medicine, Kamigyo-ku, Kyoto 602-8566, Japan; ^3^Department of Neurology, Mie University, Graduate School of Medicine, Tsu 514-8507, Japan; ^4^Department of Neurology, School of Medicine, Sapporo Medical University, S1W16, Chuo-ku, Sapporo 060-8543, Japan

## Abstract

In Alzheimer disease (AD) patient brains, the accumulation of amyloid-*β* (A*β*) peptides is associated with activated microglia. A*β* is derived from the amyloid precursor protein; two major forms of A*β*, that is, A*β*1-40 (A*β*40) and A*β*1-42 (A*β*42), exist. We previously reported that rat microglia phagocytose A*β*42, and high mobility group box protein 1 (HMGB1), a chromosomal protein, inhibits phagocytosis. In the present study, we investigated the effects of exogenous HMGB1 on rat microglial A*β*40 phagocytosis. In the presence of exogenous HMGB1, A*β*40 markedly increased in microglial cytoplasm, and the reduction of extracellular A*β*40 was inhibited. During this period, HMGB1 was colocalized with A*β*40 in the cytoplasm. Furthermore, exogenous HMGB1 inhibited the degradation of A*β*40 induced by the rat microglial cytosolic fraction. Thus, extracellular HMGB1 may internalize with A*β*40 in the microglial cytoplasm and inhibit A*β*40 degradation by microglia. This may subsequently delay A*β*40 clearance. We further confirmed that in AD brains, the parts of senile plaques surrounded by activated microglia are composed of A*β*40, and extracellular HMGB1 is deposited on these plaques. Taken together, microglial A*β* phagocytosis dysfunction may be caused by HMGB1 that accumulates extracellularly on A*β* plaques, and it may be critically involved in the pathological progression of AD.

## 1. Introduction

Alzheimer's disease (AD) is characterized by the deposition of amyloid-*β* (A*β*) plaques, accumulation of neurofibrillary tangles (NFTs), and loss of synapses and neurons in particular brain areas [1]. Experimental studies using transgenic AD mouse models have demonstrated that A*β* accelerates NFT formation [2, 3] and is closely associated with synaptic damage [4]. In contrast, A*β* reduction in the brain by A*β* immunization restores cognitive functions in transgenic AD mouse models [5–9] and also appears to slow cognitive decline in human AD patients [10]. Thus, the accumulation of A*β* may play a key role in the pathogenesis of AD [11].

A*β* is derived from the sequential proteolysis of amyloid precursor protein (APP) by *β*- and *γ*-secretases and is composed of 37–43 amino acid residues because *γ*-secretase, which is a protein complex including presenilin (PS), generates the C-terminal of A*β* with different lengths [12]. Among the variations in A*β*, A*β*1-40 (A*β*40) and A*β*1-42 (A*β*42) are the major species found in AD brains. The most predominant species deposited in A*β* plaques is A*β*42 [13], which is prone to aggregation [14] and indicates increased neurotoxicity [15]. On the other hand, A*β*40 is the major soluble species; its secretion is 10-fold more than that of A*β*42 in normal brains. A previous study demonstrated that the deposition of A*β*40 in AD brains is particularly correlated with synaptic and neuronal loss [16]. Thus, lowering the concentration of A*β*40 and A*β*42 in the brain may serve as a disease-modifying therapy for AD patients.

Activated microglia accumulate on A*β* plaques in AD brains. Although microglial accumulation was initially believed to be involved in the formation of A*β* plaques [17], experimental studies later demonstrated the ability of microglia to phagocytose A*β* peptides [18, 19]. In addition, we demonstrated microglial contribution in A*β*42 clearance using primary cultured rat microglia [20–25]. However, it has been reported that microglial dystrophy occurs in aging human brains [26], and the age-related disability of microglial A*β* phagocytosis has been demonstrated experimentally [27]. Thus, the dysfunction of microglial A*β* phagocytosis appears to be closely involved in the progression of AD pathology.

High-mobility group box protein 1 (HMGB1) is an abundant nonhistone chromosomal protein that is released from cells undergoing necrosis [28, 29]. The released HMGB1 behaves like an inflammatory mediator by acting on receptor for advanced glycation end products (RAGE) and Toll-like receptors (TLRs) 2 and 4 [30, 31]. We have previously reported that HMGB1 is extracellularly associated with A*β* plaques in AD brain and is involved in the pathogenesis of AD as an inhibitory factor against microglial A*β*42 phagocytosis by interfering with uptake [32, 33]. However, the effect of extracellular HMGB1 on the microglial phagocytosis of A*β*40, but not A*β*42, has not been elucidated. Therefore, in the present study, we analyzed rat microglial A*β*40 phagocytosis in the presence and absence of exogenous HMGB1.

## 2. Materials and Methods

### 2.1. Primary Culture of Rat Microglia and Drug Treatment

The primary culture experimental procedure was reviewed and approved by the Committee for Animal Research at Kyoto Pharmaceutical University. Primary cultured microglia (over 97% pure) were prepared, as described previously [32]. Briefly, brain tissues were isolated from newborn Wistar rats, minced, and gently dissociated by trituration in Dulbecco's modified Eagle medium (DMEM). The tissue suspension was filtered through a 50 *μ*m diameter nylon mesh into 50 mL tubes, and cells were collected by centrifugation at 200 ×g for 10 min. Cells were resuspended in DMEM with 10% fetal calf serum, 100 units/mL penicillin, and 100 *μ*g/mL streptomycin; they were then plated onto 100 mm diameter dishes at 37°C in humidified 5% CO_2_/95% air. We then harvested the floating microglia from mixed glial cultures. Microglia were transferred to 24-well plates (3.0 × 10^5^ cells/well) and were allowed to adhere at 37°C overnight; they were then treated with sterilized phosphate-buffered saline (PBS) as the vehicle or synthetic human A*β*s (A*β*40 or A*β*42; Anaspec, San Jose, CA) in the presence or absence of calf thymus-purified HMGB1 (WAKO Chemicals, Osaka, Japan). We previously demonstrated that 1 *μ*M A*β*42 for 12 h markedly phagocytosed by rat microglia [20], and 0.3 *μ*M HMGB1 inhibits the phagocytosis [32, 33]. When A*β*40 at 1–3 *μ*M were added into the culture, we could detect A*β*40 phagocytosed by rat microglia by Western blot analysis [25]. Therefore, in the present study, we adopted the concentrations at 1 *μ*M and 0.3 *μ*M for the treatments with A*β*s and HMGB1, respectively. To make the experimental conditions more accurate, we dissolved the lyophilized human A*β* peptides in distilled and sterilized water at a high concentration, and small aliquots were kept at −80°C until use. Subsequently, A*β* stock solutions were diluted using sterilized PBS, and once A*β* was thawed, no A*β* was refrozen to eliminate variance due to repeated freezing and thawing.

### 2.2. Immunocytochemistry

Twelve hours after A*β* treatment, microglia were gently rinsed three times with PBS and then fixed with 4% paraformaldehyde in 100 mM phosphate buffer (PB) for 30 min. Cells were then incubated with a mouse monoclonal antibody against A*β* (clone 6E10, 1 : 1000; Chemicon, Temecula, CA) and a rabbit polyclonal antibody against HMGB1 (1 : 1000; BD Pharmingen, San Diego, CA). The primary antibodies were followed by application of a rhodamine-labeled anti-mouse immunoglobulin (Ig)G antibody and fluorescein isothiocyanate-labeled anti-rabbit IgG antibody (each diluted 1 : 500; Molecular Probes, Eugene, OR). Furthermore, cells were incubated with Hoechst 33258 (1 : 5000; Molecular Probes) to visualize microglial nuclei. Labeled fluorescence was detected using a laser scanning confocal microscope LSM 510 (Carl Zeiss, Jena, Germany).

### 2.3. A*β* Phagocytosis and Clearance Assay by Western Blot Analysis

Twelve hours after A*β* treatment, microglia and culture media were collected and lysed with Laemmli's sample buffer and then analyzed by Western blot analysis. Briefly, samples were subjected to sodium dodecyl sulfate-polyacrylamide gel electrophoresis (SDS-PAGE; 20% polyacrylamide gels in Tricine buffer). Proteins were transferred to a polyvinylidene difluoride membrane (Millipore, Billerica, MA) by electroelution and then incubated with a mouse monoclonal antibody against A*β* (clone 6E10, 1 : 2000; Chemicon), followed by a horseradish peroxidase-linked secondary antibody against mouse IgG (1 : 1,000; Amersham, Buckinghamshire, UK). Subsequently, protein bands were detected on radiographic films (Kodak, Rochester, NY) using a chemiluminescence kit (ECL kit; Amersham). For semiquantitative analysis, radiographic films were scanned with a CCD color scanner (DuoScan, AGFA, Mortsel, Belgium) and then analyzed densitometrically using the public domain US National Institutes of Health image 1.56 program.

### 2.4. A*β* Degradation Assay

Microglia were harvested and resuspended in 100 mM Tris-HCl buffer (pH 7.5) containing 10 mM KCl, 1.5 mM MgCl_2_, and 1 mM DTT and then homogenized. After centrifugation (50,000 ×g) for 20 min at 4°C, the protein concentration of the supernatant was measured and used as the microglial cytosolic fraction. The A*β* peptide (3 *μ*M A*β*40 or A*β*42) was incubated with the microglial cytosolic fraction (final concentration of 1 mg/mL) in the presence or absence of 0.3 *μ*M HMGB1. At the time points of 0, 6, and 12 h after incubation, Laemmli's sample buffer was added, and samples were boiled at 100°C for 5 min to stop A*β* degradation. Subsequently, samples were analyzed using the antibody against A*β* (clone 6E10, 1 : 2000; Chemicon) by Western blot analysis, as described previously.

### 2.5. Immunoprecipitation

HMGB1 (1.5 *μ*g, 2.6 *μ*M) was mixed with 3 *μ*g of synthetic A*β*40 (37.5 *μ*M) in PBS. Twenty-four hours after incubation, the antibody (10 *μ*g of IgG) against HMGB1 (BD Pharmingen) or A*β* (clone 6E10; Chemicon) was added to the mixture and further incubated for 2 h at 4°C. Protein A-Sepharose (50 *μ*L of a 50% slurry) was then added, and the mixture was incubated overnight at 4°C. After centrifugation, immunoprecipitates were resuspended in Laemmli's sample buffer. Subsequently, samples were analyzed using the antibody against HMGB1 (1 : 1000; BD Pharmingen) or A*β* (clone 6E10, 1 : 2000; Chemicon) by Western blot analysis, as described previously.

### 2.6. Immunohistochemical Study Using Human AD Brain Sections

All experiments using human samples were performed in accordance with the guidelines of the ethical committees of Kyoto Pharmaceutical University. Informed consent was obtained from all subjects. For histological examination, frontal cortex tissue from a patient who was clinically and histopathologically diagnosed as human AD (age, 67 years) was used. Neuropathological assessment of AD was conducted in accordance with the criteria of the Consortium to Establish a Registry for Alzheimer's Disease (CERAD). Dissected tissue blocks were fixed in 10% formalin and transferred to a 15% sucrose solution in 100 mM PB containing 0.1% sodium azide at 4°C. The cryoprotected brain blocks were cut into 20 *μ*m sections on a cryostat, and the collected sections were stored in PBS containing 0.3% Triton X-100 (PBS-T) and 0.1% sodium azide at 4°C until use.

Immunohistochemical study was essentially performed as described previously [34]. Free-floating human brain sections were treated with 0.1% hydrogen peroxide for 30 min to quench endogenous peroxidase activity; they were then incubated with 1% goat serum to block nonspecific binding in PBS. Sections were then incubated with a mouse monoclonal antibody against A*β*40 (1 : 1000; nanoTools, Teningen, Germany) and rabbit polyclonal antibody against A*β*42 (1 : 1000; IBL, Gunma, Japan), a rabbit polyclonal antibody against A*β*40 (1 : 1000; IBL) and mouse monoclonal antibody against human leukemia antigen (HLA)-DR (1 : 50; Dako, Glostrup, Denmark), or a rabbit polyclonal antibody against A*β*42 (1 : 1000; IBL) and mouse monoclonal antibody against HLA-DR (1 : 50; Dako) in PBS-T with 0.1% sodium azide for 4 days at 4°C. After washing with PBS-T, the sections were incubated with biotinylated anti-rabbit IgG antibody (1 : 2000; Vector Laboratories, Burlingame, CA) for 2 h at room temperature. The sections were then incubated with avidin peroxidase (1 : 4000; ABC Elite Kit; Vector Laboratories) for 1 h at room temperature. Subsequently, labeling was visualized by incubation with 50 mM Tris-HCl buffer (pH 7.6) containing 0.02% 3,3′-diaminobenzidine (DAB) and 0.0045% hydrogen peroxide with nickel enhancement using 0.6% nickel ammonium sulfate, which yielded a dark blue color. In the second cycle, sections were incubated with a biotinylated anti-mouse IgG antibody (1 : 2000; Vector Laboratories) for 2 h at room temperature. The sections were then incubated with avidin peroxidase (1 : 4000; ABC Elite Kit; Vector Laboratories) for 1 h at room temperature. Subsequently, the DAB reaction was performed without nickel ammonium sulfate, which yielded a brown color.

In laser confocal microscopic analysis, human AD brain sections were treated with 1% goat serum to block nonspecific binding in PBS. Sections were then coincubated with a rabbit polyclonal antibody against A*β*40 (1 : 1000; IBL) and a mouse monoclonal antibody against HLA-DR (1 : 50; Dako) or a mouse monoclonal antibody against A*β*40 (1 : 1000; nanoTools) and a rabbit polyclonal antibody against HMGB1 (1 : 1000; BD Pharmingen) in PBS-T with 0.1% sodium azide for 4 days at 4°C. The primary antibodies were probed with Alexa Fluor 546-labeled anti-rabbit IgG antibody and Alexia Fluor 488-labeled anti-mouse IgG antibody or Alexa Fluor 546-labeled anti-mouse IgG antibody and Alexia Fluor 488-labeled anti-rabbit IgG antibody (each diluted 1 : 500; Molecular Probes). Subsequently, fluorescence was observed using a laser scanning confocal microscope LSM 510 (Carl Zeiss).

### 2.7. Statistical Evaluation

Results of the densitometric analysis are given as the mean ± standard error of mean. The statistical significance of differences was determined by analysis of variance. Further statistical analysis for *post hoc* comparisons was conducted using the Bonferroni/Dunn test (StatView, Abacus Concepts, Berkeley, CA).

## 3. Results

### 3.1. Binding of HMGB1 with A*β*40

**Figure 1:**
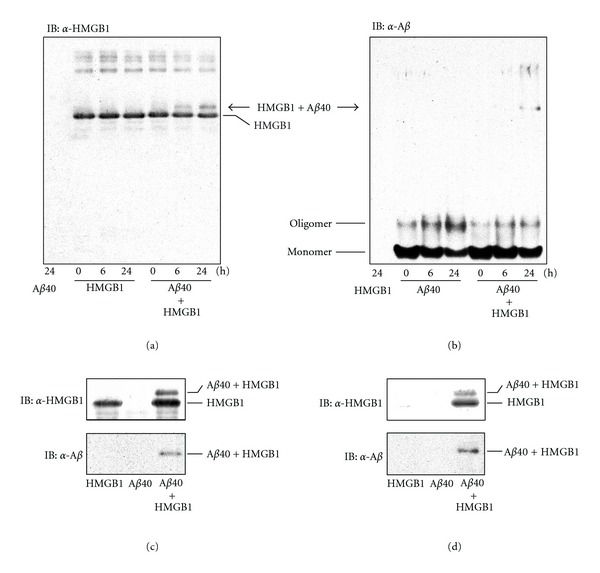
Binding affinity of HMGB1 for A*β*40. (a, b) After incubation of HMGB1, A*β*40, and the mixture of HMGB1 with A*β*40 for 0, 6, and 24 h, samples were analyzed by Western blot analysis using the anti-HMGB1 antibody (a) or anti-A*β* antibody (b). (c, d) After incubation of HMGB1, A*β*40, and the mixture of HMGB with A*β*40 for 24 h, samples were immunoprecipitated using the anti-HMGB1 antibody (c) or anti-A*β* antibody (d). The precipitates were then analyzed by Western blot analysis using the anti-HMGB1 antibody and anti-A*β* antibody.

In our previous study, we found that HMGB1 is extracellularly accumulated on A*β* plaques in AD brains and further demonstrated that HMGB1 binds to A*β*42 in *in vitro* cell-free study [33]. In the present cell-free study, we first examined the binding affinity of HMGB1 for A*β*40. Following incubation of the HMGB1 peptide alone, a 29 kDa band of HMGB1 and its high-molecular-weight aggregates was detected, while an approximately 33 kDa band (arrow in Figure 1(a)), which is believed to be a complex of HMGB1 and A*β*40, appeared as an upper band 6 h after incubation of HMGB1 and A*β*40 peptides (Figure 1(a)). Following incubation with A*β*40 (Figure 1(b)), monomers and oligomers of A*β*40 were the major components present in the absence of HMGB1 at each time point. Predictably, the 33 kDa band, which seemed to be a complex of HMGB1 and A*β*40, was detected by the addition of the HMGB1 peptide (arrow in Figure 1(b)).

To confirm the binding affinity between HMGB1 and A*β*40, we further examined immunoprecipitation using the anti-HMGB1 antibody (Figure 1(c)) or anti-A*β* antibody (Figure 1(d)). As a result, in the mixture of HMGB1 and A*β*, the complex of HMGB1 and A*β*40 (approximately 33 kDa) was immunoprecipitated with A-Sepharose-linked antibodies against HMGB1 (Figure 1(c)) or A*β* (Figure 1(d)). These results demonstrated that HMGB1 had a binding affinity for A*β*40.

### 3.2. Microglial A*β* Phagocytosis and Effect of Exogenous HMGB1

**Figure 2:**
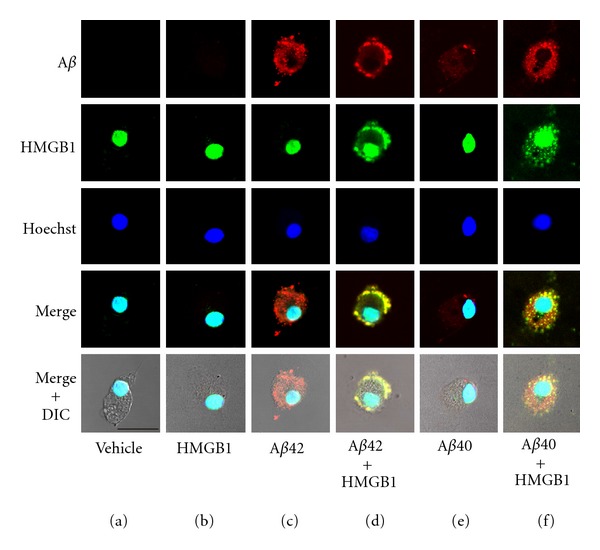
Effect of exogenous HMGB1 on microglial A*β* phagocytosis analyzed by laser confocal microscopy. Rat microglia were incubated with the vehicle (a), HMGB1 (b), A*β*42 (c), A*β*42 and HMGB1 (d), A*β*40 (e), or A*β*40 and HMGB1 (f) for 12 h. Fixed cells were further incubated with the anti-A*β* antibody (red), anti-HMGB1 antibody (green), and Hoechst 33258 (dye for nuclei; blue); they were analyzed using a laser scanning confocal microscope. DIC: differential interference contrast. Scale bar = 20 *μ*m for all panels.

We previously demonstrated that microglia markedly phagocytose A*β*42 [25], and extracellular HMGB1 inhibits phagocytosis on the cell surface [32, 33]. In the present study, we analyzed the microglial A*β*40 phagocytosis and the effects of extracellular HMGB1 on phagocytosis using laser confocal microscopy (Figure 2). Endogenous HMGB1 was detected in the nuclei of primary cultured rat microglia (Figures 2(a)–2(f), cyan). When treated with the vehicle (Figure 2(a)) or HMGB1 alone (Figure 2(b)), no A*β* immunoreactivity was detected. Consistent with previous studies, in the presence of A*β*42, microglia phagocytosed A*β*42 (Figure 2(c), red), exogenous HMGB1 was colocalized with A*β*42 on the microglial cell surface, and A*β* internalization was inhibited (Figure 2(d), yellow). When treated with A*β*40, the immunoreactivity of A*β*40 was barely detected in the microglial cytoplasm (Figure 2(e), red). Interestingly, in the presence of exogenous HMGB1, small vesicle-like immunoreactivities of A*β*40 (Figure 2(f), red) and HMGB1 (Figure 2(f), green) were markedly increased in the microglial cytoplasm, and parts of them were colocalized with each other (Figure 2(f), yellow).

### 3.3. Amounts of A*β*40 inside and outside Microglia and Effect of Exogenous HMGB1

**Figure 3:**
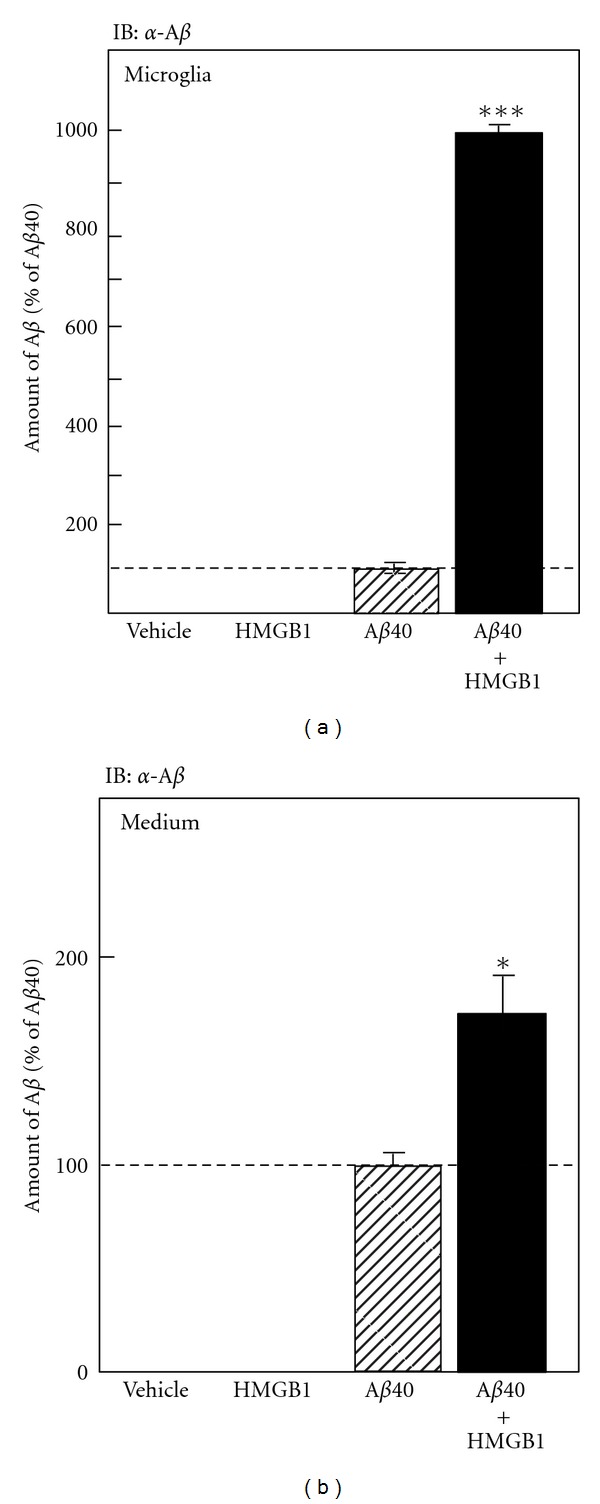
Effect of exogenous HMGB1 on microglial A*β* phagocytosis analyzed by Western blot. Rat microglia were incubated with the vehicle, HMGB1, A*β*40, or A*β*40 and HMGB1 for 12 h. Microglial cell lysate (a) and conditioned medium (b) were then subjected to Western blot analysis using the anti-A*β* antibody, and then the amounts of A*β*40 inside (a) and outside microglia (b) were semiquantitatively measured. *P < 0.05, ***P < 0.001 versus treatment with A*β*40 alone.

Twelve hours after A*β*40 treatment, microglial cell lysate and conditioned medium were collected and subjected to Western blot analysis; semiquantitative analysis was then examined to measure the concentration of A*β*40 inside (Figure 3(a)) and outside microglia (Figure 3(b)). When microglia were treated with the vehicle or exogenous HMGB1 alone, no A*β*40 immunoreactivity was detected inside them (Figure 3(a)). After treatment with A*β*40, a small amount of A*β*40 was detected inside microglia (A*β*40 phagocytosed by microglia), and this amount increased dramatically by simultaneous treatment with exogenous HMGB1 (Figure 3(a)). This result raises two possibilities: (i) extracellular HMGB1 increases microglial A*β*40 uptake, and (ii) HMGB1 inhibits the degradation of A*β*40 in the microglial cytoplasm after uptake. To address these possibilities, we measured the amount of A*β*40 in the culture medium (A*β*40 remaining outside microglia) (Figure 3(b)). After treatment with the vehicle or exogenous HMGB1 alone, no A*β* was detected in the culture medium. Twelve hours after A*β*40 treatment, the amount of A*β*40 in the culture medium significantly increased by simultaneous treatment with exogenous HMGB1. Thus, in the presence of exogenous HMGB1, the amount of A*β*40 both inside and outside microglia was much higher than that when treated with A*β*40 alone. These results suggest that exogenous HMGB1 phagocytosed by microglia inhibits the degradation of A*β*40 in the microglial cytoplasm and subsequently delays A*β* clearance by microglia.

### 3.4. A*β* Degradation with the Microglial Cytosol Fraction and Effect of Exogenous HMGB1

**Figure 4:**
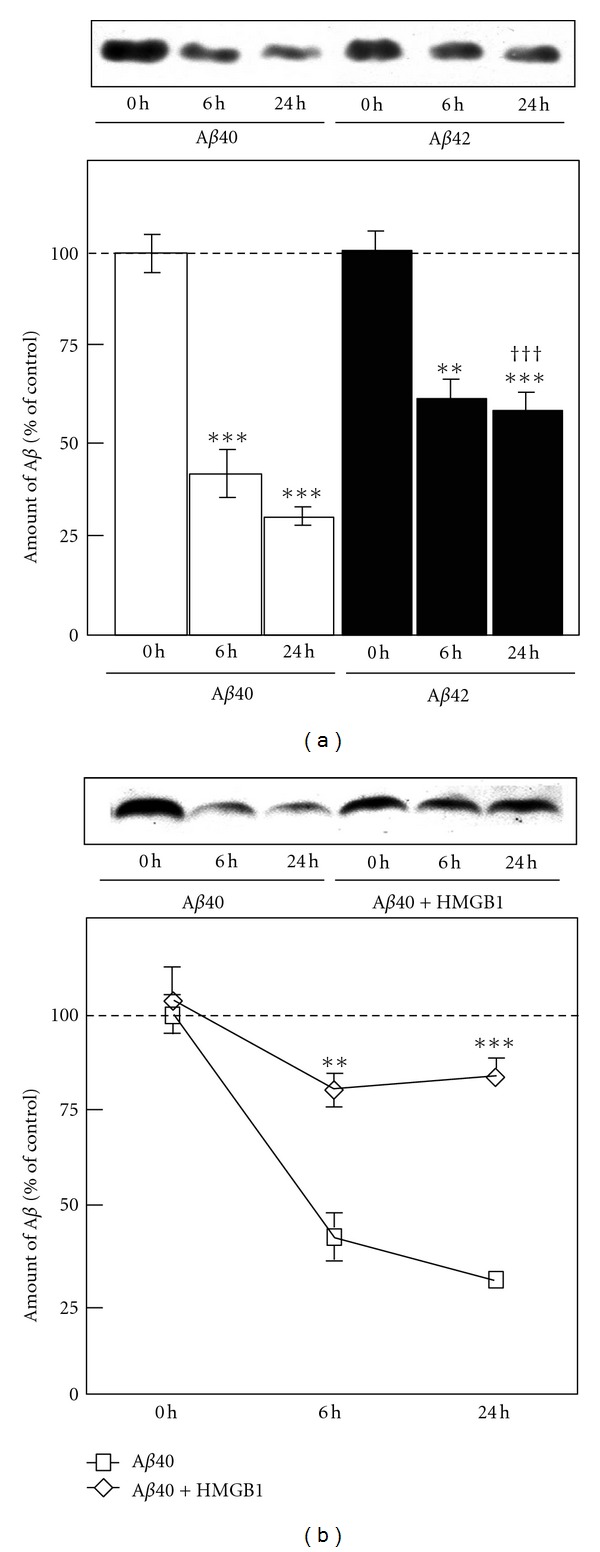
A*β* degradation with the microglial cytosolic fraction. (a) A*β*40 and A*β*42 were incubated with the rat microglial cytosolic fraction for 0, 6, and 24 h. After incubation, samples were subjected to Western blot analysis using the anti-A*β* antibody, and the amount of A*β* was semiquantitatively measured. **P < 0.01, ***P < 0.001 versus time point 0 h. ^†††^ *P* < 0.001 versus A*β*40 at time point 24 h. (b) A*β*40 and A*β*40 with HMGB1 were incubated with rat microglial cytosolic fractions for 0, 6, and 24 h. After incubation, the samples were subjected to Western blot analysis using the anti-A*β* antibody, and the amount of A*β* was semiquantitatively measured. **P < 0.01, ***P < 0.001 versus A*β*40 alone at each time point.

To confirm whether exogenous HMGB1 inhibits A*β*40 degradation in microglial cytoplasm, we prepared cytosolic fractions from rat microglia and mixed them with A*β*. Degradation of A*β*40 and A*β*42 by microglial cytosol fractions was compared (Figure 4(a)). A*β*40 and A*β*42 were gradually degraded by the addition of the microglial cytosolic fraction in a time-dependent manner. A*β*40 was degraded earlier than A*β*42 (Figure 4(a)). We next examined the effect of exogenous HMGB1 on the A*β*40 degradation induced by the microglial cytosolic fraction (Figure 4(b)). At 6 and 24 h after incubation, the degradation of A*β*40 was significantly delayed by the addition of exogenous HMGB1. Thus, this result suggests that exogenous HMGB1 phagocytosed by microglia inhibits the degradation of A*β*40 in the microglial cytoplasm.

### 3.5. Accumulation of A*β*40, A*β*42, and Microglia in AD Brains

**Figure 5:**
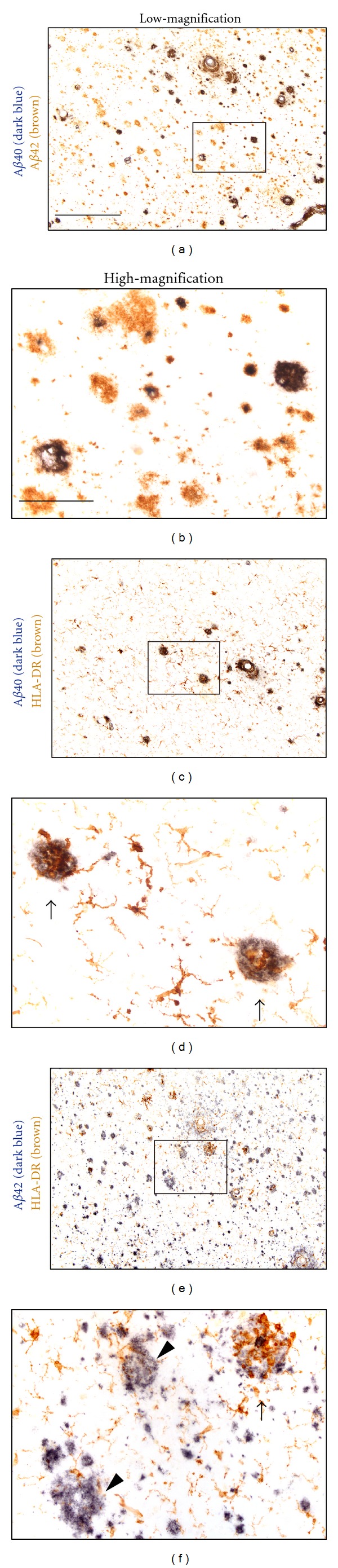
Immunohistochemical study of microglial accumulation on A*β* plaques in human AD brains. Free-floating human AD brain sections were incubated with the anti-A*β*40 specific antibody (dark blue) and anti-A*β*42 specific antibody (brown) (a, b), anti-A*β*40 specific antibody (dark blue) and anti-HLA-DR antibody (for microglial staining; brown) (c, d), and anti-A*β*42 specific antibody (dark blue) and anti-HLA-DR antibody (for microglial staining; brown). Arrows and arrow heads show marked and poor microglial accumulations, respectively. (b), (d), and (f) show high-magnification views of squared area in (a), (c), and (e), respectively. Scale bar in (a) equals 400 *μ*m for (a), (c), and (e). Scale bar in (b) equals 100 *μ*m for (b), (d), and (f).

We further investigated the localization of A*β*40 and A*β*42 in AD brains using specific antibodies (Figures 5(a) and 5(b)) and microglial accumulation on the plaques composed of A*β*40 (Figures 5(c) and 5(d)) and A*β*42 (Figures 5(e) and 5(f)). The number of A*β*40 plaques (dark blue deposits in Figure 5(a)) was lesser than that of A*β*42 plaques (brown deposits in Figure 5(a)). High-magnification photographs revealed that A*β*40 accumulated on A*β*42 plaques (Figure 5(b)). Regarding microglial accumulations (Figures 5(c)–5(f)), almost all A*β*40 plaques were markedly surrounded by activated microglia (Figure 5(c) and arrows in Figure 5(d)). Although some A*β*42 plaques were markedly accumulated by microglia (Figure 5(e) and arrow in Figure 5(f)), others were moderately or poorly surrounded by microglia (arrowheads in Figure 5(f)).

### 3.6. Accumulation of HMGB1 and Microglia on A*β*40 Plaques in AD Brains

**Figure 6:**
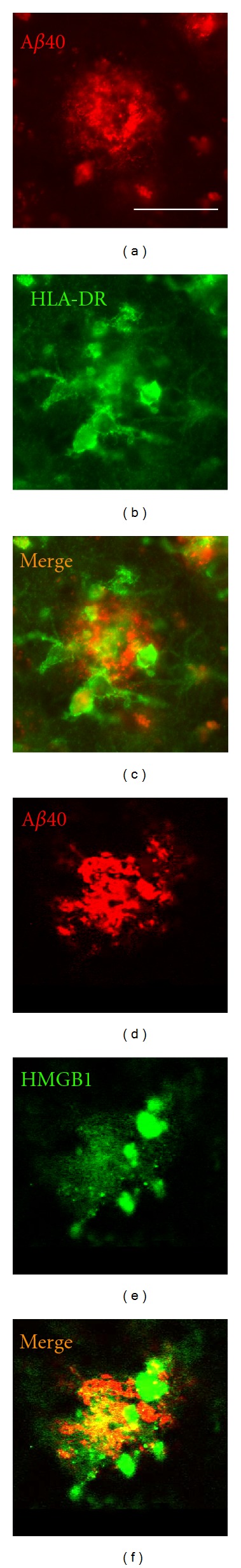
Laser confocal microscopic study on the accumulation of HMGB1 and microglia on A*β*40 plaques in human AD brains. (a–c) Free-floating human AD brain sections were incubated with the anti-A*β*40 specific antibody ((a) red) and anti-HLA-DR antibody ((b) for microglial staining, green). The merged image is indicated in (c). (d–f) Free-floating human AD brain sections were incubated with the anti-A*β*40 specific antibody ((a); red) and anti-HMGB1 antibody ((b); green). The merged image is indicated in (f). Scale bar in (a) equals 50 *μ*m for all panels.

We previously demonstrated that extracellular HMGB1 accumulates on A*β* plaques, as detected using an anti-A*β* antibody that reacts with a broad spectrum of A*β* species [33]. Therefore, in the present study, we investigated the colocalization of extracellular HMGB1 on A*β*40 plaques in AD brains using a specific anti-A*β*40 antibody. Consistent with the immunohistochemical study (Figures 5(c) and 5(d)), microglia (Figure 6(b)) markedly accumulated on A*β*40 plaques (Figure 6(a)) in AD brains (Figure 6(c)). We further demonstrated that extracellular HMGB1 (Figure 6(e)) colocalized with A*β*40 plaques (Figure 6(d)) in AD brains (Figure 6(f)).

## 4. Discussion

In studies on familial AD, mutations in the *APP*, *PS1*, and *PS2* genes have been detected, and transgenic mice models carrying these familial AD-linked mutations show enhanced A*β* production in their brains. In particular, transgenic mice carrying the *APP* mutation display characteristics that closely resemble AD, such as A*β* deposition and memory dysfunction [35, 36], and introduction of the double mutations of *PS/APP* exhibits the early onset of these pathologies [37]. Thus, all mutations are involved in A*β* generation, and the accumulation of A*β* in the brain has been strongly suggested to be the primary event driving the pathogenesis of AD. However, familial AD accounts for less than 1% of all AD cases [38]; most cases develop sporadically. Although the etiology of sporadic AD remains much more elusive than that of familial cases, neurological and pathological events in sporadic AD are essentially indistinguishable from those in familial cases. In sporadic AD, a decreased A*β* clearance rate has been reported [39].

One proposed mechanism of A*β* clearance is microglial A*β* phagocytosis [40, 41]. Reports on AD patients treated with A*β* immunization also indicate microglial contribution to A*β* clearance in human AD brains [42, 43]. However, it has been suggested that the ability of microglia to clear A*β* decreases with age and progression of AD pathology [26, 27], and it may, at least in part, account for the dysregulation of A*β* clearance in sporadic AD.

HMGB1 inhibits microglial A*β*42 phagocytosis by interfering with A*β*42 internalization [32, 33]. In the present study, we further showed that exogenous HMGB1 inhibits the degradation of A*β*40 in rat microglial cytoplasm and subsequently delays A*β*40 clearance. We demonstrated the binding affinities of HMGB1 for A*β*40 and A*β*42 [33]. A*β* contains an amino acid sequence (^18^VFFA^21^) that has been identified to be essential for aggregation and fibril formation [44]. Interestingly, HMGB1 contains a homologous motif (^16^AFFV^19^), and this sequence is thought to be critically involved in the interactions of A*β* with HMGB1 [33, 45]. Among the many peptidases that have been proposed as A*β*-degrading enzymes [46], insulin-degrading enzyme, cathepsin D, and neprilysin are the principle enzymes involved in microglia-mediated A*β* degradation [25, 47, 48]. Many cleavage sites that are the targets of microglial A*β*-degrading enzymes are located on and in the vicinity of the amino acid sequence (^18^VFFA^21^) of A*β* [46]. Therefore, we speculate that the cleavage sites of A*β* are masked by the binding of HMGB1; subsequently, the degradation of A*β*40 may be inhibited in the microglial cytoplasm. In case of A*β*42, A*β*42 itself forms high-molecular-weight fibrils during incubation [33]. Therefore, the binding of HMGB1 may stabilize A*β*42 fibril formation, and high-molecular-weight complex of HMGB1 and A*β*42 fibril may interrupt the uptake of A*β*42 by microglia. Thus, extracellular HMGB1 may serve as a chaperone protein for A*β* and inhibit microglial A*β* clearance by interrupting A*β*40 degradation and A*β*42 internalization by microglia. On the other hand, RAGE, TLR2, and TLR4 are receptors for HMGB1 [30, 31]; they are also involved in microglial A*β* phagocytosis [49, 50]. Therefore, there is a possibility that the interactions of HMGB1 with these receptors on microglia may be related to the inhibitory events on A*β*.

Consistent with a previous study [13], plaques containing A*β*42 predominantly existed in AD brains, and A*β*40 accumulated on parts of A*β*42 plaques. Despite the restricted distribution of A*β*40, almost all plaques containing A*β*40 were markedly surrounded by activated microglia. We previously reported that small oligomers formed by A*β*40 strongly induce rat microglial reactions such as cytokine production [51]. Thus, A*β*40 may play an important role in microglial activation and/or recruitment on A*β* plaques. However, we have found that the level of HMGB1 was significantly increased in AD brains [33], and extracellular HMGB1 accumulated on A*β* plaques. Therefore, in AD brains, microglial degradation of A*β*40 and uptake of A*β*42 may be inhibited by extracellular HMGB1 despite the marked accumulation of reactive microglia on A*β* plaques. Moreover, in the present study, we demonstrated that A*β*40 is more readily degraded by the microglial cytosolic fraction than A*β*42. However, in the presence of exogenous HMGB1, the degradation of A*β*40 by microglia is inhibited, and a lot of A*β*40 granules are existed in the cytoplasm of rat microglia as shown in Figure 2(f). Interestingly, numerous microglia containing A*β*40 granules, but not A*β*42, have also been detected in AD brains [52]. Thus, this event in AD brain is well replicated by the treatment with A*β*40 in the presence of extracellular HMGB1 in primary-cultured rat microglia. Results suggest that our findings in the effect of HMGB1 on rat microglia may reflect on the pathological event induced in AD brain and are expected the critical implication of extracellular HMGB1 in the progression of AD pathologies. In addition, we have postulated that the origin of extracellular HMGB1 is leakage from dead neurons during the progression of AD [32], like ischemic neurodegeneration [53]. Extracellular HMGB1 leaked from dead neurons may then accumulate on A*β* plaques through its binding affinity for A*β* in AD brains.

It has been reported that the released HMGB1 is involved in the pathologies of various inflammatory-related disease [54]. In ischemic stroke [55] and intracerebral hemorrhage especially [56], extracellular HMGB1 is suggested to exacerbate brain insult through the disruption of the blood-brain barrier (BBB), overfacilitation of microglia, and intense production of proinflammatory molecules. These studies also demonstrated that a neutralizing anti-HMGB1 monoclonal antibody and glycyrrhizin which bind to and inhibit cytokine-like activity of HMGB1 attenuate the brain insult induced by transient ischemia and intracerebral hemorrhage in rat, respectively. Therefore, there is a possibility that the neutralizing anti-HMGB1 monoclonal antibody and glycyrrhizin may bind to the extracellular HMGB1 accumulated on the A*β* plaques in the AD brain, cancel the inhibitory effects of HMGB1 on microglial A*β* phagocytosis, and then may provide novel therapeutic options for the AD treatment.

In conclusion, in the present study, we found that HMGB1 extracellularly accumulates on A*β* plaques containing A*β*40 in AD brains. We further demonstrated that HMGB1 has a binding affinity for A*β*40, and exogenous HMGB1 is internalized into rat microglial cytoplasm with A*β*40 and inhibits A*β*40 degradation. Subsequently, exogenous HMGB1 delays A*β*40 clearance in the culture medium. Thus, these results suggest that extracellular HMGB1 attenuates microglial A*β* clearance and is possibly involved in the progression of AD pathology.
